# Evaluating adult digital health literacy, 2020–2025: A systematic review

**DOI:** 10.1371/journal.pdig.0001075

**Published:** 2025-11-24

**Authors:** R. Constance Wiener, Bayan J. Abuhalimeh

**Affiliations:** 1 Department of Dental Public Health and Professional Practice, School of Dentistry, West Virginia University, Morgantown, West Virginia, United States of America; 2 Department of Restorative Dentistry, School of Dentistry, West Virginia University, Morgantown, West Virginia, United States of America; Iran University of Medical Sciences, IRAN, ISLAMIC REPUBLIC OF

## Abstract

Online/digital health literacy is important for individuals to evaluate the influence of such input in their care and consent for treatment. The purpose of this systematic review is to examine the digital health literacy level among adults in studies that used the eHealth Literacy Scale (eHEALS) as a measure of digital health literacy. The authors searched Google Scholar, PubMed, Scopus, and Web of Science for evidence following the Preferred Reporting Items for Systematic Reviews and Meta-Analysis Statement, 2020 (PRISMA). Included were articles in which the researchers evaluated the level of digital health literacy using eHEALS, were peer reviewed, written in English or in which English translation was provided, and were published between 2020–2025. There were 200 articles initially identified in the search, 180 were excluded resulting in a sample of 20 publications. EHEALS scores, with possibilities from 8-40, had a weighted mean of 24.3 (95%CI: 17.1-31.6). The lowest mean score was 12.57; and the highest mean score was 35.1. The highest eHEALS score was from a qualitative interview study. Nine other studies reported overall means ≥ 30. There were three with eHEALS scores below 20. Globally, there is a wide range of reported digital health literacy levels. It is critical that the public gains skill and confidence in digital health literacy for healthcare decisions. The results of this study provide evidence of a large range of digital health literacy.

## Author summary

There is variation in digital health literacy in global reports. With the emerging influence of the internet, it is important that the level of digital health literacy is evaluated to be able to inform healthcare policies.

## Background

Healthcare professionals use the internet, online sources, and online tools to conduct work such as disease identification, and symptom categorization. The integration of electronic health records into large networks has made it possible to accomplish care with greater clinical information than ever before. However, the general public also needs to understand the basic concepts of digital literacy as it relates to health when they conduct their own internet/online/other searches. Lack of digital health literacy impacts decision-making and the consent to treat process. For patients to be partners in their healthcare, and to provide informed consent for treatment, they need to have a basic understanding of digital technology; that is, they need digital health literacy.

It is important to know the extent of digital health literacy penetration over recent years (period prevalence). Such information can be of benefit in establishing educational needs, and policies. Previous researchers have reported the need for systematic reviews of prevalence data for the burden of disease as these syntheses better inform policy makers [1]. Similar to the high disease burden in vulnerable populations, individuals with low digital health literacy will also be at risk of harm from not understanding online health information presented, inability to judge its accuracy/reliability, and potentially releasing their personal health data during internet/online/other searches. There are blockchain-enabled methods to protect patient data and enhance decision-making on a large scale for healthcare systems [2]; however, such protections and decision-making aids are not readily available to individuals who often rely solely upon their own skills and digital knowledge for data protection and decision-making.

At present, there is no gold standard for evaluating digital health literacy in the general population. One tool that is often used was created in Canada to evaluate skills of using information technology for health and finding the best fit of electronic health programs. The eHealth Literacy Scale (eHEALS) is a short (8 items), convenient, and easy to understand tool [3]. It is presented as self-assessments of digital health literacy. It measures an individual’s knowledge, comfort, and skill for locating, analyzing, and using electronic health information [3]. Its theoretical basis is the social cognitive and self-efficacy theory and Lily theoretical model of eHealth Literacy in which it is a convergence of traditional literacy, computer literacy, health literacy, information literacy, science literacy, and media literacy [4]. It has a high level of internal consistency. The Item-scale correlations were from r = 0.51 to 0.76; test-retest reliability was stable with r = 0.40 to 0.68; principal components analysis had a single factor solution with 56% of variance [3], and the intraclass correlation coefficient was 0.49 [3].

### Purpose

Previous systematic reviews of digital literacy have primarily focused upon the type of digital literacy instruments being used by researchers [4–7]. These reviews have structured their groupings in their systematic synthesis by instrument type rather than a comparison of specific results from the use of one scale.

The purpose of this study is to examine and describe the extent/magnitude of digital health literacy in the current literature (published between 2020 and 2025) among studies in which the researchers evaluated digital health literacy with the eHEALS assessment tool as the study scope.

### Study design

This study is a systematic review of the 2020–2025 digital health literacy literature with a descriptive summary of studies as the outcome.

## Methods

### Ethics approval

The research for this study did not involve human subjects. It received acknowledgement as non-human subject research by the West Virginia University Institutional Review Board (certificate 12481).

### Search/reviewers

The two authors (RCW, BJA) were the reviewers for the study. RCW downloaded and printed publication titles from Google Scholar, PubMed, Scopus, and Web of Science for articles related to digital health literacy following the Preferred Reporting Items for Systematic Reviews and Meta-Analysis Statement, 2020 (PRISMA) [8]. (The PRISMA statement is the guiding framework for the study; S1 Checklist). The search was conducted on international journals from January 21, 2025, for articles published between 2020 and January 21, 2025. Key words used in the search were ((“AI” or “artificial intelligence”) and (“eHEALS” or “eHealth Literacy,”).

The search terms “AI” and “artificial intelligence” did not capture the intent of the researchers to identify only articles that included the prevalence of digital health literacy as measured with eHEALS and, though included in the initial search, were excluded as not having eHEALS outcome measures.

From the downloads, RCW and BJA selected only publications that included studies in which the results were reported from participants who responded to an eHEALS survey. RCW and BJA met to discuss the review process, then they reviewed each publication separately and independently. Reviews were compared. The reviewers were consistent and in agreement for the determinations of quality, bias, and numeracy on 18 of the potential 20 studies. The reviewers met to discuss a resolution of the two studies, and after discussion, the consensus was to include the studies (agreement = 100% after the resolution of these two disagreements) with agreed upon quality and bias assessment.

The selected studies were read to extract the setting, sample description, and eHEALS mean scores provided. The selected studies were then sorted by eHEALS mean scores in descending order of magnitude. The reviewers were in 100% agreement on the extraction of setting, sample description and eHEALS mean scores.

### Eligibility criteria

#### Inclusion.

Included in the review were complete, peer-reviewed international studies, written in English or in which English translation was provided, with eHEALS summative data published between 2020 and January 21, 2025. Only published and accessible studies were included. The inclusion was limited to peer-reviewed studies as these studies would have been previously vetted before publication whereas grey literature, such as theses and conference presentations, which are not peer reviewed, would have a greater potential for bias. Recent years were selected to be the most representative of the current status of digital health literacy.

#### Exclusion.

Not included in the study were letters to the editor/personal commentaries; citations only; conference abstracts; books; protocols; systematic reviews with no eHEALS comparisons; case studies; case series; theses/not peer reviewed articles; articles unable to be retrieved; articles that did not use eHEALS, or if eHEALS was used, did not report eHEALS quantitative results.

### Data extraction and synthesis

Studies were selected using the key words listed above. Duplicates were removed. The initial eligibility review consisted of evaluation of the titles and reading of abstracts for the use of eHEALS as an outcome. Eligible studies were read by the two authors. These data were extracted from the studies that were read: citation, design, number of participants, participant characteristics, setting (country), and outcome statistic of interest (eHEALS outcome).

### Quality assessment and bias tools

Two quality assessment and bias tools were used, the Critical Appraisal Skills Program (CASP, 2024) [9] and the risk of bias tool [10]. The CASP is an 11-item checklist for the appraisal of cross-sectional studies. The tool has three appraisal categories (yes; no; cannot tell). The components of the tool are summarized as 1) focused issue; 2) appropriate method; 3) appropriate selection; 4) appropriate measuring; 5) appropriate data collection; 6) adequate sample size; 7) appropriate result presentation; 8) rigorous analysis; 9) clear findings; 10) local application; 11) value. Details are provided at the CASP website [9]. The risk of bias tool has three options, yes (risk of bias), no (no apparent risk of bias) and cannot appraise. The tool is applicable for selection bias, attrition bias, missing data, and selective reporting [10]. The four domains (selection bias, attrition bias, missing data, and selective reporting) were separately scored (0 = bias, or cannot determine; 1 = no bias determined) and a summative score determined.

### Analytic method

Overall, a mixed-methods approach was used for analysis. The initial step was to use the CASP tool and risk of bias tool. The use of the tools by the reviewers (RCW, BJA) was reliable and consistent. Although reproducible by the reviewers the quality and bias analyses must be considered qualitative and subjective. Microsoft Excel, Microsoft Corporation, Version 2308 was used in determining the means and standard deviations for the CASP score, and the 4-point risk of bias determination.

The main study outcome was the magnitude of the mean eHEALS reported in the selected studies. The structure (structural grouping) plan was to organize the studies in descending order of magnitude from the highest reported mean eHEAL scores to the lowest, then determine the natural cut-points that resulted and describe the results. That is, provide a descriptive summary of the outcomes (mean eHEALS scores by study). As the populations represented by the samples in the 20 studies are heterogenous and representative of diverse socioeconomic regions and cultures and varying study designs, the heterogeneity of the studies was handled through the presentation of descriptive statistics regarding the characteristics of the samples (country, authors, sample size descriptions, and study design) used in the studies and group them by scores for the reader to observe the global trend. Additionally, a pooled synthesis of results was provided (though it should be considered with caution as a result of the diverse populations) to quantitatively summarize the eHEALS results. Overall weighted eHEALS mean and confidence intervals were determined by considering both the sample size (weight) and the score in relation to the total pooled sample. Sample size limitations precluded analytic structural grouping by study type, country/region, gender, or age of participants.

## Results

**Fig 1:**
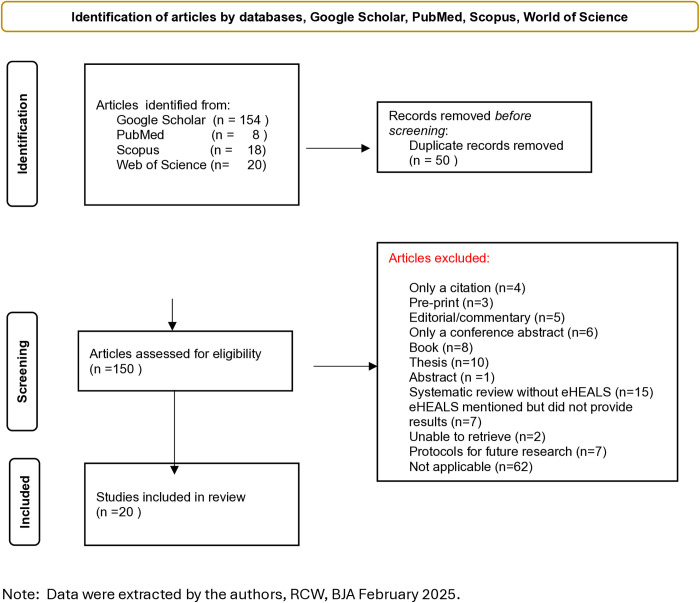
Flow chart of article selection. Note: Data were extracted by the authors, RCW, BJA January 2025.

We found 200 articles through the searches for eHEALS scores and removed 50 duplicates. There were 150 titles and abstracts screened and 130 were removed as not meeting inclusion criteria. There were 20 articles that were included in the study that used the eHEALS surveys and provided eHEALS results. The flow chart of the selection process for the 20 articles is provided in Fig 1.

The overall quality of the studies was high for the study designs selected and used. The CASP quality assessment tool with a potential score from 0 (no quality) to 11 (high quality) applied to the 20 articles resulted in an overall mean score of 9.85 (SD, 0.88, range 3). Each of the studies identified received a positive CASP score for study focus, using appropriate methods for the study design chosen, measuring the eHEALS as intended (self-report of the participant), presenting the summaries adequately and in a clear manner and were worthwhile to have been published. With the 4-point risk of bias assessment, the overall mean score was 2.2 (SD, 0.41, range 2). The score reflects multiple studies in which the criteria could not be evaluated or there were selection biases noted. Two studies had small sample sizes, though appropriate for their qualitative study design [11,12]. A third study also had a small sample size (*n* = 45) with selection of participants who were breast cancer healthcare providers attending a training session [13]. Missing data was addressed in one study [14]. Missing data was categorized as “cannot determine” in the other studies and not counted in the score. There was no indication of selective reporting or attrition bias. Sample selection was through a multistage randomization in one study [15]; multistage stratification in one study [16]; national recruitment with stratification in one study [17]; quota sampling [18,19]; purposive sampling [12]; patient recruitment [11,14,20,21]; a training session [13]; recruited and stratified by a data capture company [22]; and, self-selection from online/published calls/convenience sampling for the remaining studies. (The quality summary table is presented as Table 1 with studies listed alphabetically by first author followed by the reference number).

**Table 1:** Quality and Bias Assessment.

	Quality Assessment, CASP												Bias Assessment				
First Author, Year; Reference Number	Focus	Appropiate method	Appropiate selection	Accurate measuring	Appropiate data collection	Adequate sample size	Adequate participants	Appropiate result presentation	Rigorous analysis	Clear findings	Local application	Score summation of yes	Selection bias	Attrition bias	Missing data explained	Selective reporting	Score summation of no
Ahmed,2023; 29	Y	Y	Y	Y	Y	Y	820	Y	Y	Y	Y	**11 Y**	1 university nursing and art students; random	N	C	N	2
Al-Ruzzieh,2024; 18	Y	Y	Y	Y	Y	Y	672	Y	C	Y	Y	**10 Y**	Quota sampling	N	C	N	2
Brors,2023; 14	Y	Y	Y	Y	Y	Y	2924	Y	C	Y	Y	**10 Y**	Patient cohort from 7 hospitals	N	Y	N	2
Dallora,2024; 30	Y	Y	N	Y	Y	Y	646	Y	Y	Y	Y	**10 Y**	3 universitiesconvenience	N	C	N	2
Guo,2024; 15	Y	Y	Y	Y	Y	Y	4218	Y	Y	Y	Y	**11** **Y**	N-MultistageRandom	N	C	N	3
Griewing,2024; 13	Y	Y	N	Y	Y	N	45	Y	Y	Y	N	**9** **Y**	HealthcareProfessionals**A**t a training session	N	C	N	2
Hernández Encuentra, 2024; 22	Y	Y	Y	Y	Y	Y	800	Y	Y	Y	Y	**11** **Y**	N- 1-factorial experimental design with factor framing and factor levels of anthropomorphic framing and framing as AI along with a control group	C	C	N	2
Holgyesi,2024; 19	Y	Y	Y	Y	Y	Y	150	Y	C	Y	Y	**10** **Y**	Quota sampling	N	C	N	2
Hölgyesi,2024; 20	Y	Y	N	Y	Y	Y	1400	Y	C	Y	Y	**9** **Y**	Single center routine visit	N	C	N	2
Huiigens,2025; 11	Y	Y	N	Y	Y	N	15	Y	Y	Y	Y	**9** **Y**	Recruited from patient panel	N	C	N	2
Kopka M, 2022; 24	Y	Y	N	Y	Y	Y	494	Y	C	Y	Y	**9** **Y**	Convenience	N	C	N	2
Kubb,2022; 28	Y	Y	N	Y	Y	N	46	Y	Y	Y	Y	**9** **Y**	Leaflet distribution, social media, published call; self-selection	N	C	N	2
Kyaw,2024; 27	Y	Y	C	Y	Y	Y	434	Y	Y	Y	Y	**10** **Y**	2 cities	N	C	N	2
Lee,2025; 26	Y	Y	C	Y	Y	Y	421	Y	C	Y	C	**8** **Y**	Online recruitment from a survey panel	N	C	N	2
Li,2023; 16	Y	Y	Y	Y	Y	Y	2144	Y	C	Y	Y	**10** **Y**	N-MultistageStratified cluster random sampling method	N	C	N	3
Loeb,2023; 17	Y	Y	Y	Y	Y	Y	2904	Y	Y	Y	Y	**11** **Y**	N-National recruitment (Dynata) 4:1 stratified men to women	N	C	N	3
Sien,2024;12	Y	Y	Y	Y	Y	N	18	Y	Y	Y	Y	**10** **Y**	N-Purposive sampling	N	C	N	3
Tveter,2024; 21	Y	Y	Y	Y	Y	Y	71	Y	Y	Y	Y	**11** **Y**	Rheumatology outpatients, news paper and website recruitment	N	C	N	2
Wetzel, 2024; 25	Y	Y	C	Y	Y	Y	869	Y	Y	Y	Y	**10** **Y**	A survey was available online or as a paper and pencil version	N	C	N	2
Zrubka,2020; 23	Y	Y	N	Y	Y	Y	666	Y	C	Y	Y	**9** **Y**	Internet based survey	N	C	N	2
Overall mean (SD)												**9.85** **(0.88)**					**2.2** **(0.41)**

Note:

Results are alphabetized by first author.

Abbreviations: Y, yes; N, no; C, cannot determine; CASP, Critical Appraisal Skills Programme. CASP Cross Sectional Checklist. 2024 [8].

**Fig 2:**
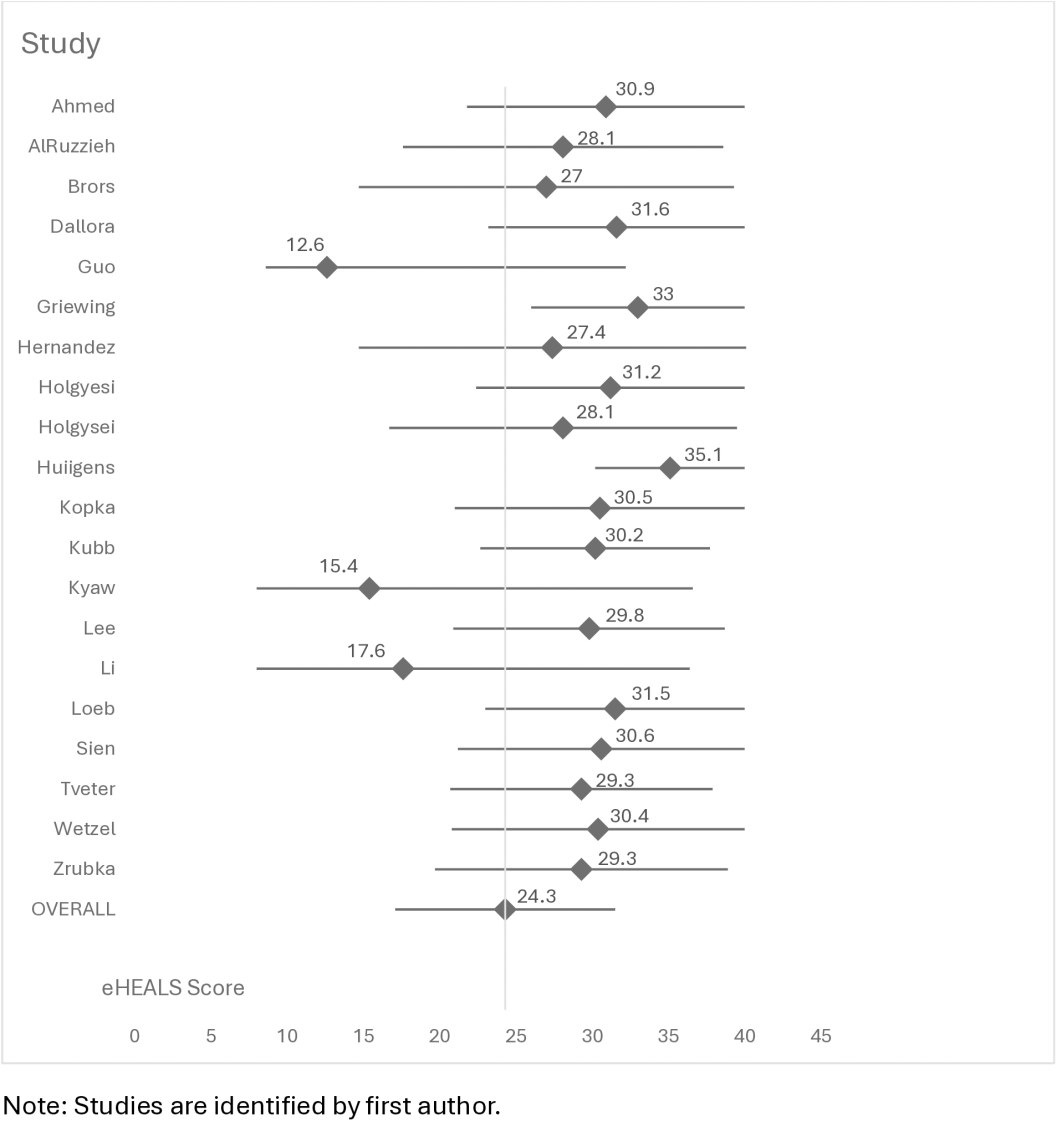
Forest Plot of Studies and Studies’ Means and 95%CI. Note: Studies are identified by first author.

The mean weighted eHEALS score was 24.3 (95%CI: 17.1-31.6) with mean individual study scores from 12.57 to 35.1 based upon potential eHEALS scores from 8-40. The highest eHEALS score was from a qualitative interview study of 15 adults in the Netherlands [11] who had an overall mean score of 35.1 (SD 8.9). Nine other studies reported overall eHEALS means ≥ 30 [12,13,17,20,24,25,28–30]. There were three with eHEALS scores below 20 [15,27]. (Fig 2).

The summary data of the studies’ eHEALS scores are presented in Table 2. They are provided in descending order arranged by each study’s eHEALS mean score (largest to smallest reported eHEALS means). Hungary was the setting for three studies [19,20,23]. China was the setting for two studies [15,16], as was the United States [17,24], Germany [13,25], and South Korea [26,27]. There were no other countries with more than one study. The other settings were Austria [28]; Canada [12]; Egypt [29]; Jordan [18]; Netherlands [11]; Norway [14]; Spain [22]; and Sweden [30]. Therefore, the populations being represented are quite heterogenous. The studies with the highest eHEALS score were conducted in Europe [1,13,20] and the U.S. [17]. The highest eHEALS scores were of adults ages 28–72 years [11]. The study with the second highest scores, included healthcare workers and students preparing to be healthcare workers [13]. The U.S. study involved adults, ages ≥40 years, recruited for a randomized trial [17], and the fifth highest scored study involved adult caregivers living with children ages 8–14 years [20].

**Table 2:** Summary of studies with eHEALS statistical means and standard deviations (SD) by descending mean eHEALS score.

Count	Ref #	Citation	Country/population	Design	Outcome eHEALS mean (SD)/categorical
1	11	Huijgens F, Kwakman P, Hillen M, van Weert J, Jaspers M, Smets E, Linn A. How Patients With Cancer Use the Internet to Search for Health Information: Scenario-Based Think-Aloud Study. JMIR infodemiology. 2025 Jan 16;5:e59625.	Netherlands, adults 28–72, n = 15Females n = 9Males n = 5Mean age 59.6	Qualitative interview study also with question-naires	Overall mean: **35.1** (SD 8.9)
2	13	Griewing S, Knitza J, Gremke N, Wallwiener M, Wagner U, Lingenfelder M, Kuhn S. Awareness and intention-to-use of digital health applications, artificial intelligence and blockchain technology in breast cancer care. Frontiers in Medicine. 2024 May 2;11:1380940.	GermanyPhysician n = 40Non-physician n = 5Working in healthcare and attending a training sessionfor breast cancer (no ages provided) Questions were for awareness and intended use of technologies	X-sectional	Overall mean: **33.04** (SD 6.61)German version of eHEALS
3	17	Loeb S, Ravenell JE, Gomez SL, Borno HT, Siu K, Nolasco TS, Byrne N, Wilson G, Griffith DM, Crocker R, Sherman R. The effect of racial concordance on patient trust in online videos about prostate cancer: a randomized clinical trial. JAMA Network Open. 2023 Jul 3;6(7):e2324395-.	United States, adults ≥ 40 years, n = 2904Females n = 1103Males n = 1801Black n = 1703 mean age 55.5White n = 1201 mean age 63.0	Randomized clinical trial, one time online survey after watching a video script	Black mean **31.8** (SD 5.7)White mean **30.9** (SD 5.8)
4	30	Dallora AL, Andersson EK, Gregory Palm B, Bohman D, Björling G, Marcinowicz L, Stjernberg L, Anderberg P. Nursing Students’ Attitudes Toward Technology: Multicenter Cross-Sectional Study. JMIR medical education. 2024 Apr 29;10:e50297.	Sweden, nursing students, ≥ 18 years, convenience sample of 3 schools, n = 646Swedish = 342,Polish = 304Females n = 555Males n = 89Mean age 23.9	X-sectional	Overall mean: 3.95 (SD 0.75)(Conversion to **31.6** SD 6)Scores <3 (<24): n = 43Scores ≥3 (≥24): n = 603
5	20	Hölgyesi Á, Luczay A, Tóth-Heyn P, Muzslay E, Világos E, Szabó AJ, Baji P, Kovács L, Gulácsi L, Zrubka Z, Péntek M. The Impact of Parental Electronic Health Literacy on Disease Management and Outcomes in Pediatric Type 1 Diabetes Mellitus: Cross-Sectional Clinical Study. JMIR Pediatrics and Parenting. 2024 Mar 20;7(1):e54807.	HungaryAdult caregivers ≥ 18 years (living with children 8–14) n = 150Females n = 120Males n = 30Mean age 42.5	X-sectional	Overall mean: **31.2** (SD 4.9)Female mean: **31.0** (SD 5.0)Male mean: **31.8** (SD 4.3)
6	29	Ahmed FM, Atia NS. Health-Related Infodemic Perception among Nursing and Non-Nursing Students: A Comparative Study. Tanta Scientific Nursing Journal. 2023 Jul 1;30(3):94–108.	Egypt, nursing students and art students, adults 18–23 years, n = 820Females n = 544Males n = 276	X-sectional	Scores 8–20 nursing: n = 13Scores 8–20 non-nursing: n = 7Scores 21–26 nursing: n = 80Scores 21–26 non-nursing: 57Scores 27–40 nursing: n = 317Scores 27–40 non-nursing: n = 346Overall mean: **30.92** (5.19)*corresponding author provided clarification
7	12	Sien SW, Kobekyaa FK, Puts M, Currie L, Hedges P, McGrenere J, Mariano C, Haase KR. Tailored Self-Management App to Support Older Adults With Cancer and Multimorbidity: Development and Usability Testing. JMIR aging. 2024 May 8;7(1):e53163.	Canada, adults ≥ 65 years, with cancer and caregivers n = 18Older adults = 15Caregivers = 3Females n = 12Males n = 6	Prototype development with Design Thinking modelQualitative study	Overall mean: **30.6** (SD 9.0)
8	24	Kopka M, Schmieding M, Rieger T, Roesler E, Balzer F, Feufel M, Determinants of Laypersons’ Trust in Medical Decision Aids: Randomized Controlled TrialJMIR Hum Factors 2022;9(2):e35219URL: https://humanfactors.jmir.org/2022/2/e35219https://doi.org/10.2196/35219.	US residents with no professional medical training.n = 494	A web-based survey	Control group eHEALS = **30.5** (SD = 4.91)Anthropomorphic group eHEALS = **30** (SD = 5.27)
9	28	Kubb C, Foran HM. Online Health Information Seeking for Self and Child: An Experimental Study of Parental Symptom Search. JMIR pediatrics and parenting. 2022 May 9;5(2):e29618.	Austria, parents, ≥ 18 years, living with a child between 0–6 years,. n = 46Females n = 40Males n = 6Mean age 33.7	Experi-mental, randomized tasks: self-seeking and proxy-seeking groups vs psychological factors	Self-seeker mean: **30.21 (**SD 3.84) n = 23Proxy-seeker mean: **30.86** (SD 3.74) n = 23
10	13	Wetzel AJ, Klemmt M, Müller R, Rieger MA, Joos S, Koch R. Only the anxious ones? Identifying characteristics of symptom checker app users: a cross-sectional survey. BMC Medical Informatics and Decision Making. 2024 Jan 23;24(1):21.https://doi.org/10.1186/s12911-024-02430-5	Germany, to investigate German citizens’ demographics, eHealth literacy, hypochondria, self-efficacy, and affinity for technology using German validated questionnaires. n = 67 per group with matching (targeted ads, social media) validated questionnaires. n = 67 per group with matching (targeted ads, social media)	X-sectional	Users of system checker applications technology:eHEALS = **30** (SD = 5.6)Nonusers:eHEALS = **30.7 (**SD = 5.4)(German version of eHEALS)
11	26	Lee S. Association Between Korean Adults’ Electronic Health Literacy and Active Participation in Health Decision-Making. CIN: Computers, Informatics, Nursing. 2025 Jan 1;43(1):e01175.	South Korea, adults ≥18, recruited from aSurvey panel froma survey companyn = 421Females n = 244Males n = 177	Correlational	Overall mean Likert: 3.72 (SD 0.57)(conversion to **29.76** SD 4.56)
12	21	Tveter AT, Varsi C, Maarnes MK, Pedersen SJ, Christensen BS, Blanck TB, Nyheim SB, Pelle T, Kjeken I. Development of the Happy Hands Self-Management App for People with Hand Osteoarthritis: Feasibility Study. JMIR Formative Research. 2024 Oct 29;8(1):e59016.	Norway, adults ≥ 40 years, with hand osteoarthritis n = 71Females n = 61Males n = 10Mean age = 64	Prototype development, intervention,Baseline and 3 month follow-up and qualitativeeHEALS measured at baseline	Overall mean: **29.3** (SD 4.4)
13	23	Zrubka Z, Brito Fernandes Ó, Baji P, Hajdu O, Kovacs L, Kringos D, Klazinga N, Gulácsi L, Brodszky V, Rencz F, Péntek M. Exploring eHealth literacy and patient-reported experiences with outpatient care in the Hungarian general adult population: cross-sectional study. Journal of medical Internet research. 2020 Aug 11;22(8):e19013.	HungaryMean age 48.9n = 666Females n = 364Males n = 302Online sample	X-sectional	Overall mean: **29.3** (SD 4.9)
14	18	Al-Ruzzieh MA, Al-Helih YM, Al-Soud Z. e-Health literacy and online health information utilization among Jordanians: A population-based study. Digital Health. 2024 Oct;10:20552076241288380.	Jordan, adults ≥18, population based quota samplen = 672Females n = 372Males n = 300Mean age 36.04	X-sectional)	Overall mean: **28.94** (SD 5.34)Scores 8–20: n = 46Scores 21–26: n = 150Scores 27–40: n = 476
15	19	Hölgyesi Á, Zrubka Z, Gulácsi L, Baji P, Haidegger T, Kozlovszky M, Weszl M, Kovács L, Péntek M. Robot-assisted surgery and artificial intelligence-based tumour diagnostics: Social preferences with a representative cross-sectional survey. BMC Medical Informatics and Decision Making. 2024 Mar 27;24(1):87.	Hungary, adults ≥40 years who imagined they needed total hip replacement. (quota sampling)Mean age 58.3n = 1400Females n = 752Males n = 648	X-sectional	Overall mean: **28.1** (SD 5.8)Female mean: **28.3** (SD 5.6)Male mean: **27.9** (SD 5.9)
16	22	Hernández Encuentra E, Robles N, Angulo-Brunet A, Cullen D, del Arco ISpanish and Catalan Versions of the eHealth Literacy Questionnaire: Translation, Cross-Cultural Adaptation, and Validation StudyJ Med Internet Res 2024;26:e49227URL: https://www.jmir.org/2024/1/e49227https://doi.org/10.2196/49227	Spain, data from Spanish-speaking and Catalan-speaking people were collected by data capture company and stratified themn = 800Spanish = 400, Catalan = 400	X-sectional	Spanish eHEALS = **27.35**(SD 8.40)Catalan eHEALS = **27.8**(SD 7.08)
17	14	Brørs G, Dalen H, Allore H, Deaton C, Fridlund B, Norman CD, Palm P, Wentzel-Larsen T, Norekvål TM. The association of electronic health literacy with behavioural and psychological coronary artery disease risk factors in patients after percutaneous coronary intervention: a 12-month follow-up study. European Heart Journal-Digital Health. 2023 Mar 1;4(2):125–35.	Norway, adults ≥18, undergoing percutaneous coronary interventionn = 2924Mean age 66 yearsFemales n = 582Males n = 2342	Prospective observational longitudinal study	Baseline mean: **27.27** (SD 6.28)12 month mean:**26.97** (SD 5.98)
18	16	Li S, Cui G, Yin Y, Xu H. Associations between health literacy, digital skill, and eHealth literacy among older Chinese adults: a cross-sectional study. Digital health. 2023 May;9:20552076231178431.	China, (n = 2144)adults ≥60 yearsurban n = 829rural n = 1315	X-sectional	Overall mean: **17.56** (9.61)Urban mean: **20.56** (10.24)Rural mean: **15.66** (8.67)
19	27	Kyaw MY, Aung MN, Koyanagi Y, Moolphate S, Aung TN, Ma HK, Lee H, Nam HK, Nam EW, Yuasa M. Socio-digital determinants of eHealth literacy and related impact on health outcomes and eHealth use in Korean older adults: community-based cross-sectional survey. JMIR aging. 2024 Aug 13;7(1):e56061.	South Korea, community dwelling adults ≥65 yearsn = 434	X-sectional	Overall mean: **15.4** (SD 10.8)Scores 8-15.9: n = 289Scores 16-31.9: n = 48Scores 32–40: n = 97
20	15	Guo Y, Hong Z, Cao C, Cao W, Chen R, Yan J, Hu Z, Bai Z. Urban-Rural Differences in the Association of eHealth Literacy With Medication Adherence Among Older People With Frailty and Prefrailty: Cross-Sectional Study. JMIR Public Health and Surveillance. 2024 Sep 11;10:e54467.	China, frail urban (n = 2316) and rural (n = 1902)adults ≥60 years without cognitive impairment (total = 4218)	X-sectional	Overall mean: **12.57** (SD 10)Urban mean: **14.48** (SD 11.35)Rural mean: **10.23** (SD 7.41)Mean was cut point to define high score:High urban score n = 620;rural n = 77High overall n = 797*corresponding author provided clarifications and research means.

## Discussion

In this systematic review of health literacy, 20 studies were found to have used eHEALS and to have provided eHEALS score means. The 20 studies were of high quality with a mean CASP of 9.85 of a potential maximum of 11. The 20 studies had a mean risk of bias of 2.2 of a potential maximum of 4. The primary risk of bias low score was associated with the reviewers being unable to determine how missing data was managed. EHEALS scores in this systematic review of the literature from 2020-2025 varied from an overall mean of 12.57 (SD 10) to 35.1 (SD 8.9) on a scale of 8 to 40; with a weighted (pooled) mean of 24.3 (95%CI: 17.1-31.6). The pooling of the heterogenous studies which included diverse socioeconomic regions and independent cultures suggests that the global value should be considered with caution. This quantitative value is provided as a summary descriptive value of the global trend for this specific, comprehensive collection of studies that evaluated participants with eHEALS scores. The intent is to provide a baseline/reference from which future researchers could use as a coarse/crude benchmark as a comparator study. This pooled estimate of the studies is not a true global mean, nor should it be considered as representing all global populations (particularly unstudied populations/cultures/geographic regions, or countries). The pooled results are also not applicable to cross-cultural comparisons nor policy recommendations. Other researchers have noted that the prevalence of digital health literacy can vary among studies [even with the same tool] as studies often use different covariates in addition to examining differing populations; and therefore, it may be inappropriate to combine the studies for a global determination of prevalence [1]. However, these same authors recognize that rigorous reviews and data analysis can provide benefit to policy makers and other readers [1]. Also, prevalence systematic reviews were described as an emerging methodology in evidence synthesis [1]. Therefore, the summary of eHEALS scores, settings, samples, and currency are strengths of the study in providing a perspective of digital health literacy levels. In this study of articles across the globe, there is an indication of a need for increased digital health literacy as half (*n* = 10) of the studies had overall eHEAL score means < 30 which is reflective of relatively low digital health literacy. The accelerated use of the internet, social media, and associated online search methodologies intersects with the global population’s ability for use. Many people are being left behind. The International telecommunication union (ITU) of the United Nations reported 32% of the world’s 8.2 billion population do not own a mobile phone [31], despite in 2020, there were 14.02 billion mobile devices and in 2025, the projection is to be18.22 billion [32]. Owning a mobile phone does not imply digital literacy, as is indicated in the results of the selected articles in this study, but ownership does provide a greater opportunity to access such knowledge. Understanding the information selected, knowing what resources are available, and identifying quality are among the aspects of digital health literacy that need to be addressed.

### Strengths

This study has several strengths. Although an exact number is not available, the data sources, Google Scholar, PubMed, Scopus, and Web of Science, have access to approximately eight million health-related articles. (A search on 2025 July 17 indicated 8,090,000 results were available on the key word, “health.”) In addition to the available data, specific outcomes were selected so that each article was compared based on the same criteria. Study quality analysis benefited from the use of the CASP appraisal checklist.

### Limitations

The study does have limitations. The results are based on the reliance of the validity and accuracy of one scale, the eHEALS. EHEALS is concise and accurate in what it measures although the technology is advancing, and newer measures will need to be implemented to fill the gap with respect to new search algorithms and the impact of AI.

Qualitative coding and theme analyses could have improved the analysis for this study. Although the data were structured and grouped by eHEALS scores, population heterogeneity likely is influencing results. For example, the three studies with the lowest scores were from one region; however, these were also studies conducted with older adults. Another limitation is that the results are based only upon studies written in English, or in which English translations were provided. Another consideration is the lack of how missing data were addressed. This is an important limitation for any study on prevalence as the unknown data could strongly affect the results in either direction. Missing data is a systematic consideration as to selection bias in cross-sectional studies or selection and attrition bias in prospective studies. Recruitment methodologies were provided in the studies, and several used convenience samples which could impact the results.

### Implications for future research

There are several areas that warrant continuing research concerning digital health literacy, specifically to measure knowledge, comfort, and skill for locating, analyzing, and using electronic health information [3]. For example, future researchers may wish to evaluate which of the key factors of eHEALS drives literacy scores. As the internet is continually evolving with different features and functions, particularly the emerging number of search algorithms, eHEALS may not be the appropriate framework to evaluate digital health literacy in the future. With the rapid growth of AI-healthcare (from AI-generated behavioral therapy to AI-generated patient information) robust measures will be needed to bridge the gap for future health literacy research. In the very near future, AI-generated diagnoses, AI-decision-making models, AI-assistants for medical intake, and secure and transparent data sharing with tamper-proof data to avoid data breaches will be criteria that patients will need to navigate and have a high literacy level about [2]. It will be crucial to be able to identify if a system is vulnerable, or inappropriately capturing protected health information, and to identify trustworthy sources of information and protected health information storage, if sharing the data is appropriate. The prevalence of these skills will need new methods and valid tests to determine what educational focus is needed and who has the greatest needs for such assistance. The digital literacy landscape is changing rapidly, and many people will be left behind and left vulnerable to bad actors. Future research and interventions are needed to meet these challenges.

## Conclusion

It is critical that the public gains skill and confidence in digital health literacy for healthcare decisions. Already, online platforms are available for patients to use in tele-dentistry, telemedicine, and tele-pharmacy for scheduling, prescriptions, contacting practices, and during visits. Individuals with limited digital health literacy have barriers to care when faced with such applications. The results of this study provide evidence that there is a wide range of digital health literacy scores and the need for improvement.

## Supporting information

S1 Checklist eHEALS Prisma Checklist. (DOCX)
